# Acute disseminated encephalomyelitis with bilateral optic neuritis following ChAdOx1 COVID-19 vaccination

**DOI:** 10.1186/s12883-022-02575-8

**Published:** 2022-02-12

**Authors:** Sai A. Nagaratnam, Alex C. Ferdi, John Leaney, Raymond Lam Kwong Lee, Yun Tae Hwang, Robert Heard

**Affiliations:** 1 Department of Neurology, Gosford Hospital, Gosford, NSW Australia; 2 The University of Sydney, Save Sight Institute, Sydney, NSW Australia; 3 Sydney Eye Surgeons, Sydney, NSW Australia; 4 Department of Radiology, Gosford Hospital, Gosford, NSW Australia; 5 Brain and Mind Centre, The University of Sydney and Gosford Hospital, Gosford, NSW Australia; 6 The University of Sydney, Sydney, NSW Australia

**Keywords:** Acute disseminated encephalomyelitis, Oxford Astra Zeneca coronavirus disease 2019 vaccine, Severe acute respiratory syndrome coronavirus 2, Demyelination, Case report

## Abstract

**Background:**

Acute disseminated encephalomyelitis (ADEM) is a rare immune-mediated inflammatory demyelinating disease of the central nervous system. We report a case of ADEM presenting with bilateral optic neuritis temporally associated with the ChAdOx1 vaccine against SARS-COVID19 virus.

**Case presentation:**

A 36-year-old female presented with bilateral optic neuritis following her first dose of the ChAdOx1 vaccine. Initial MRI Brain showed evidence of demyelination within the subcortical white matter, with no radiological involvement of the optic nerves. Visual evoked potentials were consistent with bilateral optic neuritis which was confirmed radiologically on follow up MRI. She was treated with intravenous steroids with improvement both in symptoms and radiological appearance. A pseudo-relapse occurred which was treated with a further course of intravenous steroids followed by an oral taper. The clinical, radiological and serological results were most consistent with diagnosis of ADEM.

**Conclusions:**

ADEM is an exceedingly rare complication of ChAdOx1 vaccine despite millions of doses. While it is imperative clinicians remain aware of neurological complications of vaccines, the importance of vaccination to control a pandemic should not be undermined.

**Supplementary Information:**

The online version contains supplementary material available at 10.1186/s12883-022-02575-8.

## Background

Acute disseminated encephalomyelitis (ADEM) is an immune-mediated demyelinating disease of the central nervous system [1]. It is a rare entity, with an estimated annual incidence of 0.23 to 0.4 per 100,000 children and 0.26 per 100,000 adults [2, 3]. ADEM often occurs following an antigenic challenge such as a viral illness or vaccination although it is considered unusual following vaccination, and only 5% of patients with ADEM are estimated to have received a vaccine during the preceding month [4].

The virus responsible for the current global pandemic, SARS-CoV-2 infection has been associated with ADEM as well [5]. Since the vaccination efforts to combat the pandemic began, there have also been number of published reports of possible ADEM following SARS-CoV-2 vaccination: three related to ChAdOx1 (AstraZeneca, Oxford, United Kingdom), a non-replicating viral vector vaccine; two related to Sinovac (Vero Cells, Beijing Institute of Biological Products Co., Ltd., Beijing, China), an inactivated SARS-CoV-2 vaccine, and one following Cominarty (Pfizer-BioMTech, New York, New York, USA), an mRNA vaccine [6–11]. There have been more cases of ADEM possibly related to ChAdOx1 vaccines that have been reported to authorities. Here, we present a case of probable ADEM presenting with bilateral optic neuritis occurring following administration of the ChAdOx1 vaccine.

## Case presentation

A 36-year-old woman presented to her local emergency department with a 2 day history of a right sided headache, photophobia and blurred vision in the right eye, 14 days after her first dose of ChAdOx1 vaccine. At this time, the neurological examination, including visual acuity, performed in the emergency department was reportedly normal. Her D-dimer and platelet count were 0.74 mg/L (< 0.5 mg/L) and 258 × 10^9^/L (150–400 × 10^9^/L) respectively. CT venography showed no evidence of venous sinus thrombosis and thrombosis with thrombocytopenia syndrome was considered unlikely.

She returned to the emergency department 2 days later with bilateral visual impairment and subjective colour desaturation, painful eye movements and fatigue. She did not report any weakness, sensory disturbances or bowel or bladder incontinence. Visual acuity was 6/15 (-2) on the right and 6/30 on the left which did not correct with pinhole. There was no relative afferent pupillary defect or asymmetric red desaturation. There was full range of eye movements without nystagmus or diplopia, although the eye movements themselves were painful. The rest of the upper and lower limb examination was unremarkable. She was admitted to the hospital on this occasion.

She had no significant personal medical history, including previous episodes of weakness or sensory changes and was not a migraineur. She had no known contact with COVID-19 positive cases, and no respiratory symptoms, hence a SARS-CoV-2 polymerase chain reaction test was not performed, as per the local testing protocol at that time. Her family history was notable for mother with multiple sclerosis and the patient had a normal MRI brain 5 years prior, prompted by her mother’s diagnosis.

Biochemical analysis, summarised in Table 1, did not reveal any significant abnormalities.

**Table 1:** Biochemical and serological results

Biochemistry and serology results	Result	Normal Range
*Platelet count*	225 × 10^9^/L	150- 400 × 10^9^/L
*Vitamin D*	60 nmol/L	50 -140 nmol/L
*Folate*	29.1 nmol/L	7 – 46.4 nmol/L
*Vitamin B12*	309 pmol/L	138- 652 pmol/L
*Beta 2 glycoprotein antibodies*	6 U/ml	< 20 U/ml
*Cardiolipin IgG*	< 3 U/ml	< 20 U/ml
*Cardiolipin 1gM*	2 U/ml	< 20 U/ml
*Anti nuclear antibodies*	Not detected	
*Extractable nuclear antibodies*	Not detected	
*Anti-neutrophil cytoplasmic antibodies*	Not detected	
*Serum aquaporin 4 antibodies*	Negative	
*CSF aquaporin 4 antibodies*	Negative	
*Serum myelin oligodendrocyte glycoprotein antibody*	Negative	
*CSF PCR testing—N meningitidis DNA, S pneumoniae DNA, Herpes simplex virus type 1 DNA, Herpes simplex virus type 2 DNA, Human parechovirus RNA, VZV DNA, Enterovirus RNA*	Not detected	
*CSF Glucose*	4.8 mmol/L	2.8 – 4.5 mmol/L
*CSF Protein*	0.4 g/L	0.19 – 0.56 g/L
*CSF White cells*	59 × 10^6^/L (99% mononuclear)	< 5 × 10^6^/L
*CSF Red cells*	3 × 10^6^/L	< 1 × 10^6^/L
*CSF culture*	Negative	
*CSF IgG*	0.06 g/L	< 0.03 g/L
*Serum IgG*	12.4 g/L	7- 16 g/L

Cerebrospinal fluid analysis performed on day 2 of admission showed a normal protein of 0.4 g/L (0.19 – 0.56 g/L), glucose of 4.8 mmol/L (2.8 – 4.5 mmol/L) with pleocytosis (white cell count 59 × 10^6^/L) (< 5 × 10^6^/L). CSF IgG was 0.06 g/L (< 0.03 g/L) with serum IgG of 12.4 g/L (7.0 – 16 g/L), and oligoclonal IgG bands were present, suggestive of intrathecal IgG synthesis. Serum and CSF aquaporin 4 antibodies were negative. Serum myelin oligodendrocyte glycoprotein antibody (MOG) was negative. Visual evoked potentials performed on Day 1 were unrecordable from the left eye and delayed on the right, consistent with demyelinating pathology involving the anterior visual pathways bilaterally but more severe on the left.

**Fig. 1:**
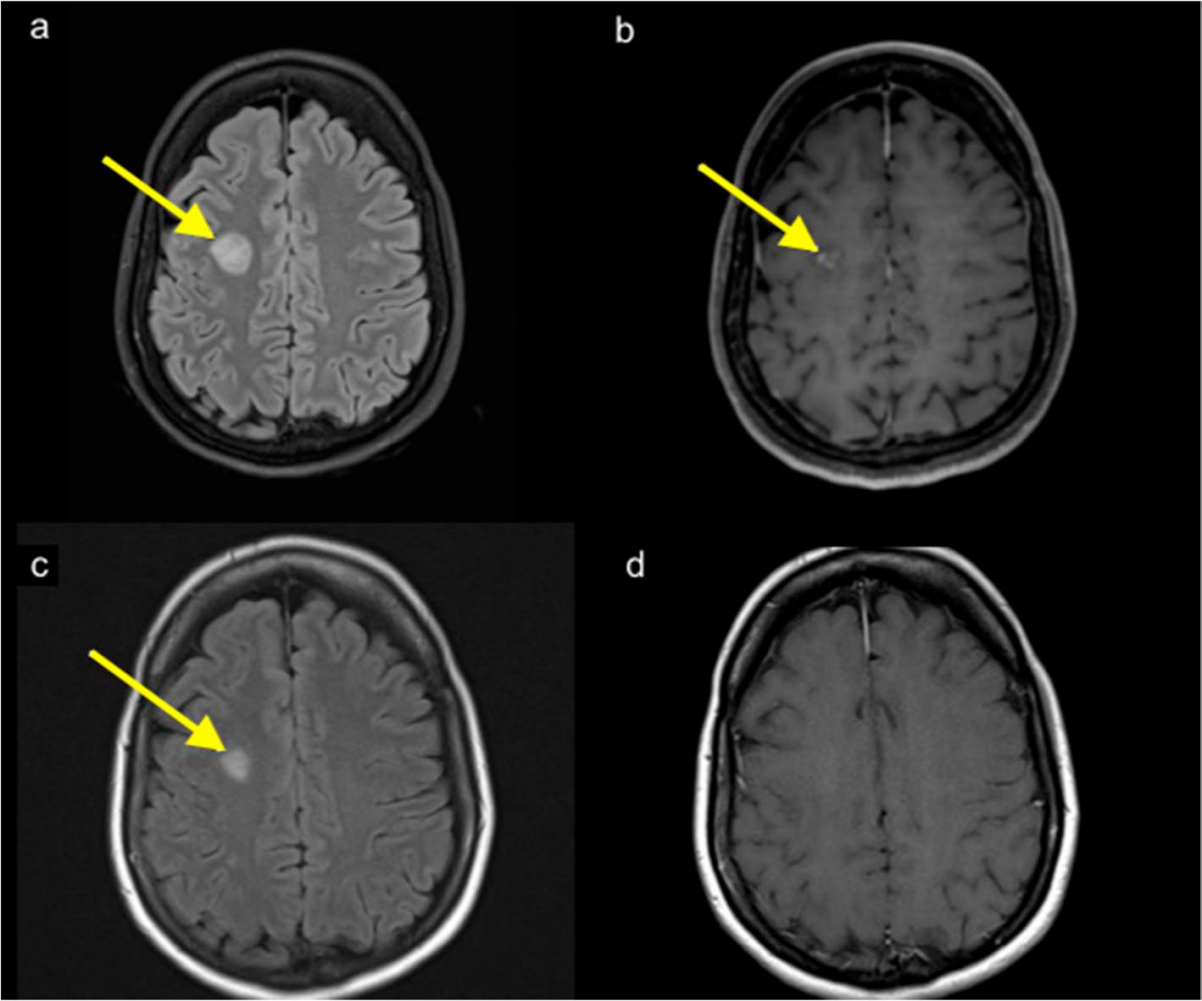
MRI Brain. **a.** Hyperintense FLAIR signal white matter lesion in the right frontal lobe (arrow). **b** Internal punctate enhancement of the right frontal lobe lesion (arrow). **c** Lesion shown in Fig. 1a reduced in size after 2 weeks (arrow). **d** Lesion no longer demonstrated abnormal enhancement

3 T MRI brain (Skyra, Siemens, Germany) also performed on Day 2 showed multiple T2/ FLAIR hyperintense lesions involving the subcortical white matter, posterior limb of bilateral internal capsules, pons and left middle cerebellar peduncle. The largest lesion was in the right frontal centrum semiovale measuring 17 × 17 mm with multiple internal punctate foci of gadolinium contrast enhancement (Fig. 1a, b). There was no callosal involvement. Notably, there was no definite abnormal signal or enhancement of optic nerves. MRI of the spine showed no evidence of demyelinating disease.

**Fig. 2:**
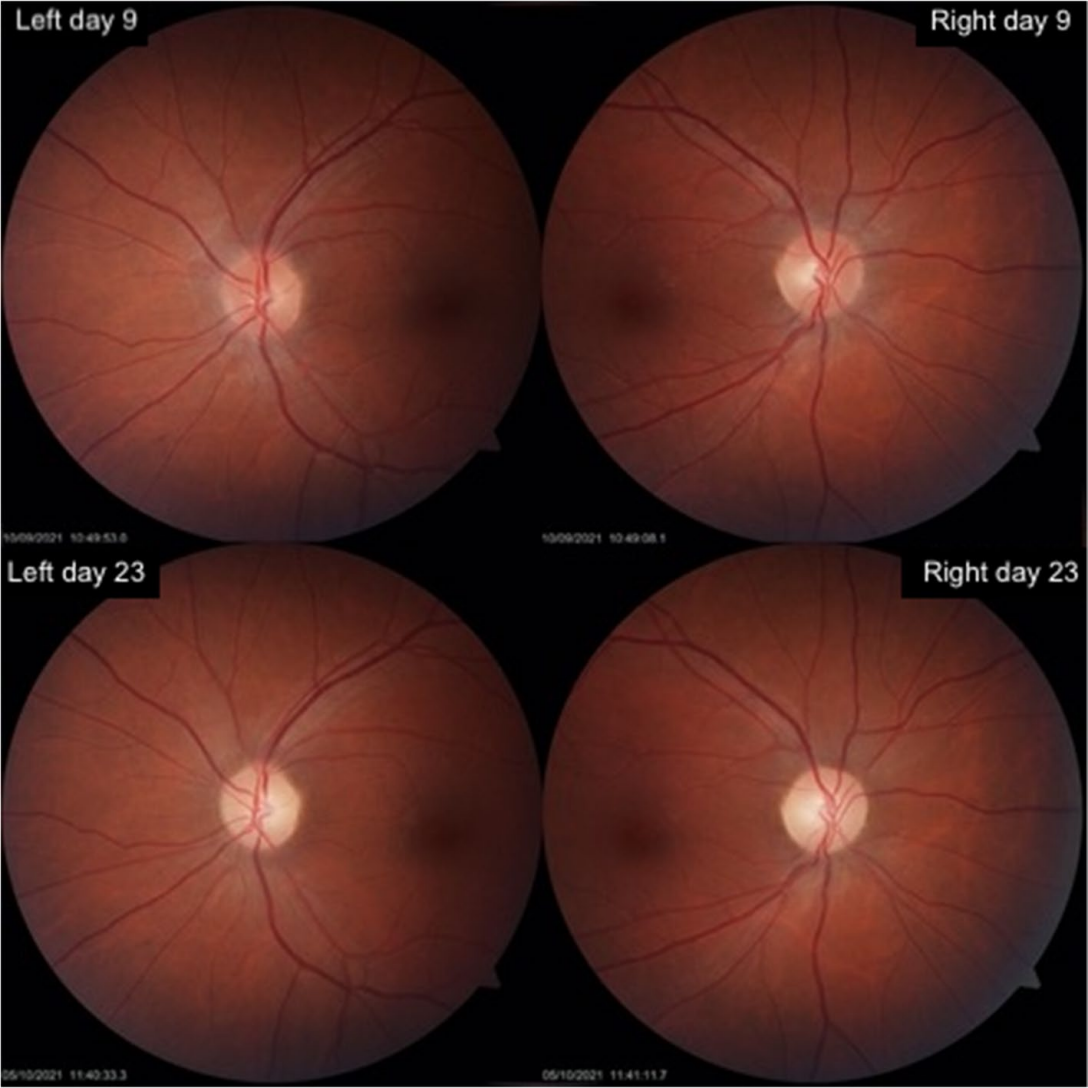
Photos of the optic nerves of the patient. Left and right optic nerves day 9 after symptom onset did not demonstrate any swelling or atrophy. Day 23 after symptom onset shows pallor bilaterally

Ophthalmology examination on Day 8 found visual acuity of 6/5 (20/16) in the right eye and 6/9 (20/30) in the left. Colour vision was depressed in both eyes with 12/17 Ishihara plates identifiable with the right eye and 10/17 with the left. There was no relative afferent pupillary defect detectable. The patient’s optic discs appeared healthy with no swelling or atrophy (Fig. 2). There was no concurrent anterior segment or retinal disease. Formal visual fields were assessed with Humphrey Field Analyzer (Carl Zeiss Meditec, Dublin, CA, USA), which demonstrated largely intact fields in both eyes, with only a few mild scattered non-specific deficits (Supplementary file 1). Optical coherence tomography (CIRRUS™ HD-OCT 5000, Carl Zeiss Meditec, Dublin, CA, USA) showed mild swelling of her retinal nerve fibre layer bilaterally, measuring a total thickness of 111 μm on the right and 108 μm on the left (Supplementary file 2).

Based on these results, we concluded that the diagnosis that would best fit the clinical scenario and the investigation results was ADEM presenting with optic neuritis. She received 3 daily doses of 1000 mg of intravenous methylprednisone with marked improvement in vision and discharged home with plans for close outpatient follow up but without tapering regime of oral steroids.

On day 15, the patient reported worsening of vision in both eyes, worse in the left. Examination revealed further reduction in visual acuity in the left eye of 6/180 (20/200) and subtle reduction in the right eye of 6/6 (20/20). The colour vision was reduced to 0/17 Ishihara plates in the right eye and could not be assessed in the left. A dense relative afferent pupillary defect was present in the left eye. Optic nerves remained normal clinically with no swelling or atrophy. Loss of visual acuity in the left eye resulted in a sensory exotropia with poor fixation that precluded visual field or optical coherence tomography testing. Visual field testing of the right eye demonstrated severe depression with an inferior altitudinal defect (Supplementary file 1). Although there was no significant change in retinal nerve fibre layer thickness detectable in the right eye, the left increased by 12 μm (Supplementary file 2).

A repeat MRI Brain on day 17 from initial presentation, 2 weeks after the initial MRI showed significant reduction in size of the multiple T2/ FLAIR hyperintense lesions throughout the subcortical white matter. The right frontal centrum semiovale lesion reduced in size to 12 × 10 mm with contrast enhancement no longer present (Fig. 1c, d). No interval development of new lesions was evident. However, there was now abnormal signal and enhancement of both optic nerves but more prominent on the left, consistent with optic neuritis. These radiological findings mirrored the results of the visually evoked potentials performed during the first admission.

A further course of 3 doses of 1000 mg daily of intravenous methylprednisone improved her visual symptoms and she was discharged home on 50 mg daily of oral prednisolone with a tapering plan.

23 days after her initial symptoms her clinical picture had significantly improved. Her visual acuity was near baseline: right 6/5 (20/16), left 6/6 (20/20) with full Ishihara colour vision bilaterally. Both optic nerves demonstrated pallor (Fig. 2). Her visual field testing was near full on the right and although the left was significantly improved, a dense inferior altitudinal defect remained (Supplementary file 1). Her optical coherence tomography imaging revealed the retinal nerve fibre layer swelling had improved bilaterally (Supplementary file 2) but her ganglion cell layer thickness of the retina was thinned by 15 μm in each eye suggesting irreversible optic nerve damage (Supplementary file 3).

**Fig. 3:**
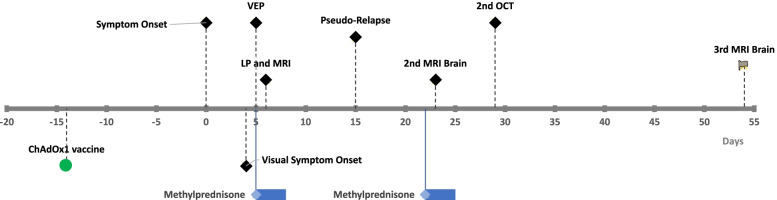
Timeline of symptom onset and investigations in relation to ChAdOx1 vaccination

Repeat MRI Brain 6 weeks after symptom onset showed further improvement in radiographic appearance of the lesions with no contrast enhancement to suggest active inflammation. Visual evoked potentials at 3 months showed improvement in response bilaterally with responses being detectable on the left, where they were previously absent. There were no new symptoms to suggest a clinical relapse, consistent with a monophasic illness. A summary of the timeline of symptoms and investigations is presented in Fig. 3.

## Discussion and conclusions

Our patient presented with clinical, imaging and CSF features consistent with ADEM, with her symptoms starting 14 days following her first dose of ChAdOx1 vaccine. This is the first report of ADEM associated with, or following the ChAdOx1 vaccine, presenting with bilateral optic neuritis.

ADEM is defined as a polysymptomatic event which includes optic neuritis and encephalopathy, that may manifest as behavioural change, confusion, irritability, alteration in consciousness and lethargy [1]. While no diagnostic criteria exists for adults, in children it requires the presence of both encephalopathy and multifocal central nervous system involvement (Table 2) [1]. Optic neuritis is often bilateral and is much more common (~ 25%) as a presenting symptom of both post-viral and post- vaccine ADEM, as in our patient, than in multiple sclerosis where it is relatively rare [12–14]. Evidence of inflammation within the cerebrospinal fluid with pleocytosis and lack of intrathecal oligoclonal band synthesis are more likely in ADEM than in multiple sclerosis, although there is some overlap, as seen in this case [15]. CSF oligoclonal bands were detected in our patient and two other cases of ChAdOx1 associated ADEM [7]. Important differentials in ADEM include multiple sclerosis, neuromyelitis optica spectrum disorder (NMOSD) and myelin oligodendrocyte glycoprotein antibody disease (MOGAD) which we have done our best to exclude [1]. A relapse, as in our case, may occur within 4 weeks of steroid tapering or within 3 months of the sentinel event, but is defined as a part of the monophasic illness [4].

**Table 2:** Definition of monophasic ADEM. From consensus definition for paediatric demyelinating disorders by Krupp et. al [1]

Clinical event with presumed inflammatory or demyelinating cause, with acute of subacute onset that affects multifocal areas of the CNS.
Clinical presentation must be polysymptomatic and must include encephalopathy
Encephalopathy is defined as one or more of the following.
-Behavioural change e.g. confusion, irritability
-Alteration in consciousness e.g. lethargy, coma
Clinical event followed by clinical and/ or radiological improvement with possibility of residual deficits
No history of clinical episodes with features of a prior demyelinating event

MRI findings suggestive of ADEM include multifocal punctate to large flocculent T2/FLAIR hyperintensities that are often bilateral and asymmetrical, involving peripheral grey and white matter junction. Tumefactive, mass-like lesions are also possible. The brainstem and posterior fossa can be involved, but usually not the callososeptal interface. Enhancement pattern is variable—punctate, ring, incomplete ring, peripheral enhancement have all been reported and absence of enhancement does not exclude the diagnosis [16]. Findings can overlap with those of multiple sclerosis which has predilection for the corpus callosum and periventricular white matter as well as the subcortical U fibres [16]. Active demyelination plaques in multiple sclerosis typically demonstrate incomplete ring enhancement. In our patient the internal punctate foci of enhancement are not typical of an active plaque but is more suggestive of ADEM. This, in combination with absence of lesions in the corpus callosum or lesions involving the subcortical U fibres renders ADEM a more likely radiological differential diagnosis than multiple sclerosis.

Specifically for the ChAdOx1 vaccine, there have been approximately 11.6 million doses administered just in Australia as of 20^th^ September 2021 [17]; many more doses have been administered globally. Transverse myelitis, another disorder of central nervous system demyelination, has already been associated with SARS-CoV-2 vaccinations, with at least two cases being reported following ChAdOx1 vaccination [18–20]. Shared antigenic epitopes between a virus or vaccine protein and host myelin protein result in T and subsequent B-cell activation from antigen presentation is thought to initiate an immune inflammatory reaction and demyelination in both conditions [21].

There have been 3 other cases of ChAdOx1 associated ADEM in the literature (Table 3) [6–8]. Symptom onset occurred between 7–14 days from vaccine administration. Our case is somewhat different from the previous cases of ChAdOx1 associated ADEM – the patient presented with bilateral optic neuritis and there was no evidence of spinal cord involvement, which was a feature of the other reported cases.

**Table 3:** Reported cases of ChAdOx1 associated ADEM

Case	Age/ Sex	Days post ChAdOx1 vaccine at symptom onset	Optic Nerve involvement	Spinal cord involvement	CSF oligoclonal bands	Encephalitis	Treatment
Our case	36, Female	14	Yes	No	Yes	Yes (Lethargy)	Intravenous methylprednisone, oral corticosteroids
Permezal, et al. [6]	63, Male	12	No	Yes	Yes	Yes (Poorly responsive)	Intravenous methylprednisone, plasmapheresis
Rinaldi et al. [7]	45, Male	12	No	Yes	Yes	No	Intravenous methylprednisone, oral corticosteroids
Mumoli et al. [8]	45, Male	7	No	Yes	No	No	Intravenous methylprednisone

While this individual had a family history of multiple sclerosis affecting a first degree relative, there is paucity of data on significance of MS family history in adult onset ADE, and no definite association in the paediatric population has been identified [22, 23]. It remains to be seen in a family history of MS confers increased risk of developing ADEM.

Another interesting aspect of this case is that despite clear initial symptoms suggesting optic nerve involvement, the first MRI did not demonstrate any degree of optic nerve enhancement despite the clearly abnormal VEP, confirming its higher sensitivity than MRI for optic neuritis and its utility still in twenty-first century [24].

Corticosteroids are the mainstay of therapy for ADEM, however plasma exchange or intravenous immunoglobulin may be considered for refractory cases [4]. Complete recovery is the expected course in the majority of post-vaccination cases of ADEM [25].

While ADEM does occur following vaccination, ADEM is a vanishingly rare complication following ChAdOx1 vaccine. ADEM should be considered in patients presenting with visual disturbances following ChAdOx1 vaccination, even without other long tract findings. It is imperative as clinicians to be aware and remain vigilant for potential rare complications of vaccinations such as ADEM to improve our understanding of immune system and ADEM. Our report, by highlighting the rarity and reversibility of ADEM, help to emphasise the safety of vaccines that prevent serious illness and death, particularly in a global pandemic.

## Supplementary Information

**Additional file 1.** Humphrey visual field tests of the left and right eye. Dots represent visual field points tested with normal field. Shaded or black squares denote partially or severely depressed field respectively at this test point. The left eye visual acuity was very poor and the eye deviated outward on September 22^nd^ so could not complete the test. By September 30^th^ the left eye vision had much improved and the now recordable but still depressed visual field is seen. In the right eye, a severe visual field deficit is seen on September 22^nd^ which almost completely resolves by September 30^th^.

**Additional file 2.** Optic coherence tomography of each eye analysing optic nerve retinal nerve fibre layer thickness. Both eyes were mildly thickened on presentation. On relapse the left nerve fibre layer thickened significantly whereas the right eye was largely unchanged. On final review the nerve fibre layer swelling had improved bilaterally.

**Additional file 3.** Optical coherence tomography of each eye analysing ganglion cell layer thickness at the macula. Both eyes demonstrate sequential thinning of the ganglion cell layer over each visit suggesting irreversible optic nerve damage.

## Data Availability

Not applicable.

## Abbreviations

ADEM: Acute disseminated encephalomyelitis
