# Supplementary figures and images for: Acute disseminated encephalomyelitis with bilateral optic neuritis following ChAdOx1 COVID-19 vaccination
Source: BMC Neurol. 2022 Feb 12;22:54. doi: 10.1186/s12883-022-02575-8 (PMC8840677; doi:10.1186/s12883-022-02575-8)

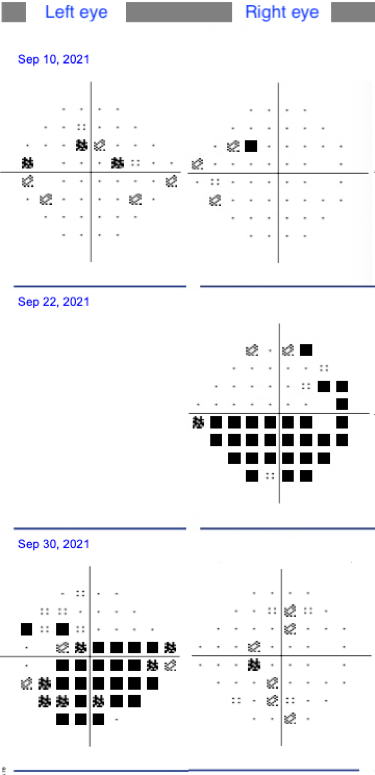

Supplement: Supplementary file 1 — Additional file 1. Humphrey visual field tests of the left and right eye. Dots represent visual field points tested with normal field. Shaded or black squares denote partially or severely depressed field respectively at this test point. The left eye visual acuity was very poor and the eye deviated outward on September 22nd so could not complete the test. By September 30th the left eye vision had much improved and the now recordable but still depressed visual field is seen. In the right eye, a severe visual field deficit is seen on September 22nd which almost completely resolves by September 30th. [file 12883_2022_2575_MOESM1_ESM.png]

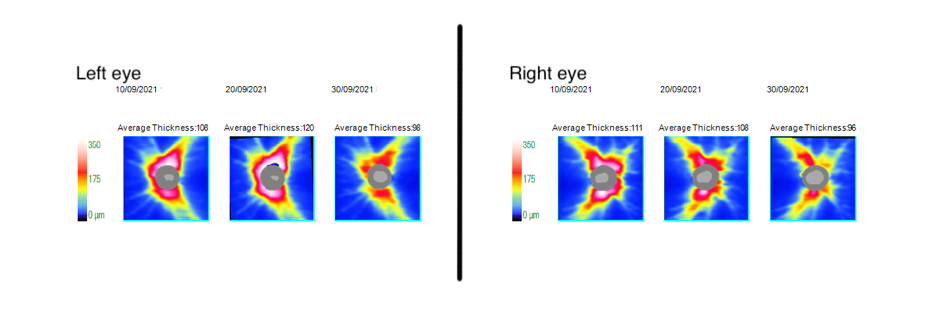

Supplement: Supplementary file 2 — Additional file 2. Optic coherence tomography of each eye analysing optic nerve retinal nerve fibre layer thickness. Both eyes were mildly thickened on presentation. On relapse the left nerve fibre layer thickened significantly whereas the right eye was largely unchanged. On final review the nerve fibre layer swelling had improved bilaterally. [file 12883_2022_2575_MOESM2_ESM.png]

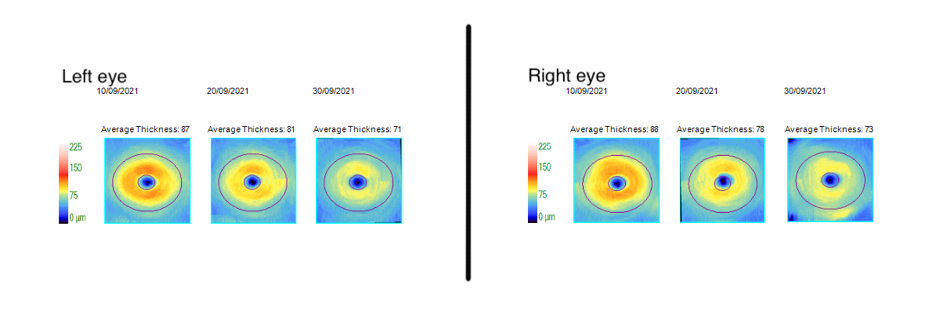

Supplement: Supplementary file 3 — Additional file 3. Optical coherence tomography of each eye analysing ganglion cell layer thickness at the macula. Both eyes demonstrate sequential thinning of the ganglion cell layer over each visit suggesting irreversible optic nerve damage. [file 12883_2022_2575_MOESM3_ESM.png]
